# The influence of demographic, health and psychosocial factors on patient uptake of the English NHS diabetes prevention programme

**DOI:** 10.1186/s12913-023-09195-z

**Published:** 2023-04-11

**Authors:** David Reeves, Adrine Ablitt Woodham, David French, Peter Bower, Fiona Holland, Evangelos Kontopantelis, Sarah Cotterill

**Affiliations:** 1 National Institute for Health Research School for Primary Care Research, School of Health Sciences, Faculty of Biology, Medicine and Health, The University of Manchester, Manchester, UK; 2 Centre for Biostatistics, School of Health Sciences, Faculty of Biology, Medicine and Health, The University of Manchester, Manchester, UK; 3 Manchester Centre for Health Psychology, Division of Psychology & Mental Health, School of Health Sciences, University of Manchester, Manchester, UK; 4 Centre for Primary Care and Health Services Research, School of Health Sciences, NIHR ARC Greater Manchester, The University of Manchester, Manchester, UK

**Keywords:** Pre-diabetes, Diabetes, Non-diabetic hyperglycaemia, NHS-DPP, Diabetes prevention programme, Risk factors, Psychosocial

## Abstract

**Background:**

The prevention of type 2 diabetes (T2DM) is a major concern for health services around the world. The English NHS Diabetes Prevention Programme (NHS-DPP) offers a group face-to-face behaviour change intervention, based around exercise and diet, to adults with non-diabetic hyperglycaemia (NDH), referred from primary care. Previous analysis of the first 100,000 referrals revealed just over half of those referred to the NHS-DPP took up a place. This study aimed to identify the demographic, health and psychosocial factors associated with NHS-DPP uptake to help inform the development of interventions to improve uptake and address inequities between population groups.

**Methods:**

Drawing on the Behavioral Model of Health Services Utilization we developed a survey questionnaire to collect data on a wide range of demographic, health and psychosocial factors that might influence uptake of the NHS-DPP. We distributed this questionnaire to a cross-sectional random sample of 597 patients referred to the NHS-DPP across 17 general practices, chosen for variation. Multivariable regression analysis was used to identify factors associated with NHS-DPP uptake.

**Results:**

325 out of 597 questionnaires were completed (54%). Only a third of responders took up the offer of a place. The best performing model for uptake (AUC = 0.78) consisted of four factors: older age; beliefs concerning personal vulnerability to T2DM; self-efficacy for reducing T2DM risk; and the efficacy of the NHS-DPP. After accounting for these, demographic and health-related factors played only a minor role.

**Conclusion:**

Unlike fixed demographic characteristics, psychosocial perceptions may be amenable to change. NHS-DPP uptake rates may be improved by targeting the beliefs of patients about their risk of developing T2DM, their ability to carry out and sustain behaviours to reduce this risk, and the efficacy of the NHS-DPP in providing the necessary understanding and skills required. The recently introduced digital version of the NHS DPP could help address the even lower uptake amongst younger adults. Such changes could facilitate proportional access from across different demographic strata.

**Supplementary Information:**

The online version contains supplementary material available at 10.1186/s12913-023-09195-z.

## Background

The prevention of type 2 diabetes mellitus (T2DM) is a major concern for health agencies around the world [1, 2]. Progression to T2DM can be prevented or delayed by behaviour change such as healthier diet, weight loss, increasing physical activity and stopping smoking [3–5]. T2DM prevention programmes identify those at high risk then offer support to change behaviour. There is strong evidence that such programmes can reduce the incidence of T2DM [6–11]. The NHS Diabetes Prevention Programme (NHS-DPP) in England offers a behaviour change intervention, based around exercise and diet, to adults diagnosed with non-diabetic hyperglycaemia (NDH), referred to the programme by a primary care professional. The programme comprises an individual initial assessment, followed by at least 16 scheduled group meetings.

The uptake of T2DM prevention programmes is often low, reducing the population-level effectiveness of the intervention and resulting in considerable resource waste [9]. Of the first 100,000 referrals to the NHS-DPP from primary care, just over half attended the initial assessment [12]. Information on the characteristics of non-participants is limited to age, sex, socioeconomic status and HbA1c or fasting plasma glucose.

Attendees of the initial assessment have ethnicity, employment, disability, smoking status, weight and BMI also recorded. This data reveals subsequent attrition to be higher amongst men, younger age-groups, those living in the most deprived areas, ethnic minorities, those in employment and those reporting a disability [12, 13]. However, whilst it may be expected that similar factors influence initial uptake – or not - of the referral, direct evidence is required. In addition, many of these relationships are weak and unlikely to account for the wide extent of non-engagement. A better understanding of what influences uptake of the programme, especially modifiable factors, is needed to help inform adjustments to programme content or delivery and the development of methods for increasing engagement. This could also provide a deeper understanding of the nature of programme access inequalities.

We aimed to gain a deeper understanding of the factors associated with NHS-DPP uptake, by undertaking a survey of people with NDH who had been referred to the programme and comparing the characteristics of those who enrolled with those who did not. The survey collected data on a wide range of potential explanatory factors, including demographic characteristics, health and health literacy, family and community context, and psychosocial factors such as beliefs, values, attitudes and knowledge about T2DM, prevention and related topics [14].

We adopted Andersen’s Behavioral Model of Health Services Utilization (BMHSU) as a framework for informing and structuring the survey content [15, 16]. The BMHSU has previously been used in T2DM self-management to explore the characteristics facilitating or impeding health services utilization [17]. This model views health behaviours as being influenced by a combination of enabling, predisposing, and health need factors, both individual and contextual. Enabling factors encompass individual, family, and wider resources, such as income, transport, and service availability. Predisposing factors include personal characteristics such as age, sex and ethnicity, along with contextual factors such as community demographics and psychosocial factors concerning attitudes and knowledge, social norms, and perceived control [14, 16]. Health need factors include functional capacity and general state of health, plus contextual factors such as community morbidity levels, that impact on care-seeking and treatment-adherence [16].

We used this framework to help identify, select and organize a range of sociodemographic characteristics, beliefs and context known or hypothesised to be associated with progression of T2DM and utilisation of diabetes prevention services [12, 18, 19]. However, for analytical and interpretative purposes, we found it expedient to cross-classify the factors into demographic, health need and psychosocial subgroups and our findings are presented primarily in line with these groupings.

### Aims

To undertake a questionnaire survey of people with NDH offered the NHS-DPP by their GP in order to:

1. Quantify the associations between a wide range of patient demographic, health and psychosocial characteristics and uptake of the NHS-DPP.
2. Explore the relative contribution of demographic, health and psychosocial factors in explaining individual uptake of the NHS-DPP.

## Methods

### Participants

#### Practices

To ensure diversity, general (family) practices (GP practices) were recruited from Greater Manchester, a largely deprived northern urban area, and from the Thames Valley and South Midlands region, a mainly affluent southern rural area. In each area the study was supported by the local Primary Care Network (PCN), which promoted the study to practices in their area and facilitated recruitment amongst those expressing interest in participating.

Practices had to have been be actively referring patients to the NHS-DPP for at least 15 months, with the principal method of referral being via a letter from the GP to a patient’s home address. The issuing of a letter constituted the referral: GPs were not permitted to send patient contact details or other data directly to the DPP provider. Upon receipt of the letter, patients interested in enquiring further were required to contact the NHS-DPP provider for further information and possible sign-up to a course. At the time of the study, all NHS-DPP courses nationally were delivered face-to-face in a group setting at community sites; digital-based courses only became available later.

#### Patients

Patients eligible for our study were those who had been referred in the above manner by their GP practice to the NHS-DPP. In addition, the referral must have been within the prior 3 to 15 months; the lower limit ensured sufficient time for the patient to have made a decision about participation in the programme; the upper limit reduced potential recall problems.

### Procedure

At each practice the pool of all patients eligible for our study was first identified via a search for an NHS-DPP referral code in their electronic record. Next, a random sample was selected from this pool. Each selected patient was sent a questionnaire and information sheet plus a covering letter from the practice. A cost-free phone number and email address was provided for any questions patients might have. Informed consent was given by return of a completed questionnaire. Fieldwork took place March to December 2019. To encourage a high response each patient received up to three mailshots and one telephone call. We contacted respondents where possible to collect any missing data values. Participants were offered £25 in shopping vouchers for returning a completed questionnaire.

### Materials

The questionnaire was designed to capture a wide range of patient-level factors potentially related to a decision to participate in the NHS-DPP or not. To encourage a high response rate, the form was restricted to four sides of A4. Using the BMHSU theoretical model of enabling, predisposing and health need factors as a framework, we conducted a literature review to identify and select the topic areas to be addressed within the instrument and to then populate these with specific question items.

Literature searches in PubMed and Google Scholar were undertaken in December 2017. A qualitative thematic synthesis approach was taken to achieve conceptual saturation in identified areas, in preference to an exhaustive search for all existent evidence [20]. To obtain broad conceptual coverage, we initially searched for quantitative and qualitative systematic reviews of lifestyle change programmes. We then conducted a further search for individual papers examining differences between participants and non-participants of self-management programmes.

Five relevant systematic reviews were identified, examining individual factors associated with: uptake and completion of lifestyle change in patients with cardiovascular conditions (n = 32 quantitative studies [21]), lifestyle change to reduce vascular risk (n = 33 qualitative studies [22]), women’s adherence to physical activity (n = 35 qualitative studies [23]), uptake of and adherence to an exercise referral (n = 20 quantitative studies [24]) and improving uptake and adherence in cardiac rehabilitation (n = 23 quantitative studies [25]). The search for papers comparing participants and non-participants of self-management programmes identified 8 papers (5 randomised controlled trials and 3 other designs) covering T2DM [17], chronic disease [26], asthma [27, 28], chronic obstructive pulmonary disease [29], arthritis [30], older people [31], and a workplace scheme [31].

From the searches we identified 44 topic areas and populated each topic with specific questions from the reviewed literature and targeted searches of wider literature when necessary. We categorised question items into demographic, health, or psychosocial subsets and also according to whether they were predisposing, enabling or health need factors.

Finally, we undertook an iterative process of assessment to reduce the number of topics and related question items to a manageable size for the survey, selecting between overlapping topics and removing low-frequency and less crucial items. The final set of explanatory factors is summarised below.

#### Demographic factors

These included age, gender and ethnicity, all conceptualised as pre-disposing an individual to participate or not, also living situation (alone, with one other person, or with more than one other), employment (working, not working), and area Index of Multiple Deprivation (IMD) [32] as enabling factors. The IMD is a UK Government small-area composite deprivation score calculated as the weighted sum of seven indices relating to income, employment, education, health, living environment, access to services and crime. IMD was conceptualised as a contextual enabling factor.

#### Health factors

Having a close relative or friend with T2DM, previous participation in a health-promotion group, and health literacy [17] were hypothesised as relevant pre-disposing factors. The latter used the Single Item Literacy Screener (SILS) health literacy scale [33]. Practical barriers to programme access were regarded as dis-enabling of health-related behaviour, so we included six items relating to: language and culture; other health problems; time; money; mobility and disability (Table A1). We combined these into a six-item “barriers” scale, where each reported barrier scored 1 point [34]. General health (the single General Health item from the Rand SF-36) [35] and mental health (MHI-5 mental health scale, 5 items [36]) were included as perceived need factors.

#### Psychosocial factors

We selected six psychosocial factors from the topic list. Generalised self-efficacy [37]; perceptions about the NHS-DPP; attitudes to the risks associated with T2DM; perceived ability to reduce risk; motivation to reduce risk; and belief in taking an active health role. Generalised self-efficacy was assessed using a pre-existing validated 4-item scale [38]. To evaluate the other five factors we generated a set of 14 question items, drawing on published items where possible, including two previously validated items from the Patient Activation Measure (Table B1) [39].

### Item validation

The survey questionnaire was populated, as far as possible, with questions used extensively in other studies with established validity. Where this was not possible, questions were developed by the research team. All questions were piloted, assessed and refined for face validity in two meetings with the DIPLOMA patient and public involvement group. This iterative process ensured the questions were readable and clear, while maintaining relevance to the factors in the theoretical model.

The outcome variable, uptake, was defined as attendance at the NHS-DPP initial assessment appointment. The questionnaire is provided in Additional File 2.

### Sample size

To incorporate practice variability a multi-site survey was planned. Taking study resource constraints into consideration, power analysis indicated that 20 practices with 11 patients from each (N = 220) would provide 78% power to detect a moderate to large association (standardised beta = 0.4) between a continuous factor and DPP uptake, assuming a moderate intra-cluster correlation of 0.1 [40]. Survey response rates in primary care can be low, therefore we set a distribution target of 30 referred patients per practice (N = 600).

### Statistical analysis

Descriptive statistics were used to characterise the sample in relation to uptake of the NHS-DPP and the various patient characteristics. Confirmatory and exploratory factor analysis, used to evaluate the psychosocial scales, is described in Additional File 1 Supplement B.

We used univariable logistic regression to investigate the ability of each explanatory factor to account for patient uptake of the NHS-DPP and produced an overall model using backwards stepwise multivariable regression of all variables, with a removal criteria of p > 0.05. GP practice was treated as a random effect. We excluded patients who claimed to have received no invitation onto the NHS-DPP, as the focus was on factors associated with a conscious decision to participate or not. Factors measured on continuous (e.g. age) or ordinal (e.g. general health) scales were analysed as continuous. To evaluate the relative contribution of each subset of factors (demographic, health and psychosocial) to uptake we conducted further multivariable logistic regression, analysing each subset as a group and also in combination with the other subsets. These models included all the factors within each subset, regardless of evidence or not for a univariate relationship with uptake.

To graphically depict the performance of each model we derived propensity scores, representing an individual’s probability of NHS-DPP uptake given their set of personal characteristics [41]. We assessed model performance according to the McKelvey & Zaviana pseudo r-squared, the area under the receiver operating curve (AUC) and the Akaike Information Criterion (AIC).

## Results

A total of 17 GP practices were recruited: 12 from Greater Manchester and 5 from the Thames Valley and South Midlands region. Recruitment halted at 17 practices (3 less than the target of 20) due to time constraints. At each recruited practice a random selection of 30 patients referred to the NHS-DPP were sent our survey, except for two practices with totals of 20 and 37 referrals all of whom were included. To make up the shortfall relative to the target sample size of 600, 3 practices randomly selected a further 30 referrals which brought the total up to 597 patients.

A total of 325 completed questionnaires were returned of the 597 distributed (54%). Rates of missing data were low, the highest for any single question item being 3% (11 cases) and the majority less than 1%.

### Demographic and health characteristics

Respondents had a mean age of 65 years, almost half were male (50.5%) and 81% self-identified as white (Table 1). 31.6% were in work, 27.4% had higher qualifications (advanced “A” levels or above) and 20% lived alone. 24.2% had previously participated in a health promotion group, 81.9% had a family member or acquaintance with T2DM, 77.5% never or rarely needed help reading health information and 25.5% rated their general health as excellent or very good.

**Table 1:** Demographic and health characteristics of the survey sample

	All (n = 325)	Participant^a^ (n = 115)	Non-participant (n = 112)	No recall of referral (n = 98)
Gender (%)				
Male	50.5% (162)	52.7% (59)	51.8% (58)	46.4% (45)
Female	49.5% (159)	47.3% (53)	48.2% (54)	53.6% (52)
Age (mean (SD))	64.8 (14.3)	67.1 (11.3)	63.6 (11.7)	63.6 (14.3)
IMD decile^b^ (mean (SD)) (higher score is less deprived)	5.4 (3.0)	6.4 (3.0)	5.0 (3.2)	4.6 (3.0)
Employment				
Working (full, part-time or self-employed)	31.8% (103)	27.2% (31)	37.5% (42)	30.6% (30)
Not working	68.2% (221)	72.8% (83)	62.5% (70)	69.4% (68)
Ethnic group				
White	80.9% (262)	81.6% (93)	82.1% (92)	78.6% (77)
Other	19.1% (62)	18.4% (21)	17.9% (20)	21.4% (21)
Highest qualification				
None, GCSE,O,CSE	57.3% (185)	49.1% (56)	60.7% (68)	63.9% (62)
A level, Degree, other	42.7% (138)	50.9% (58)	39.3% (44)	36.1% (35)
Living situation				
Live alone	20.1% (65)	22.1% (25)	17.9% (20)	20.4% (20)
With one other	55.4% (179)	60.2% (68)	53.6% (60)	52.0% (51)
With two or more	24.5% (79)	17.7% (20)	28.6% (32)	27.6% (27)
Ever joined another health group				
Yes	24.1% (78)	26.1% (30)	21.6% (24)	24.5% (24)
No	75.9% (246)	73.9% (85)	78.4% (87)	75.5% (74)
Known someone with diabetes				
Yes family	39.5% (128)	40.9% (47)	36.6% (41)	41.2% (40)
Yes non-family	42.3% (137)	45.2% (52)	44.6% (50)	36.1% (35)
No or unsure	18.2% (59)	13.9% (16)	18.8% (21)	22.7% (22)
General Health				
Excellent or v. good	25.6% (83)	27.0% (31)	25.9% (29)	23.7% (23)
Good	36.1% (117)	36.5% (42)	38.4% (43)	33.0% (32)
Fair or poor	38.3% (124)	36.5% (42)	35.7% (40)	43.3% (42)
Help on health literacy				
Never	53.7% (173)	51.3% (58)	58.9% (66)	50.5% (49)
Rarely	23.6% (76)	27.4% (31)	17.9% (20)	25.8% (25)
Sometimes, often or always	22.7% (73)	21.2% (24)	23.2% (26)	23.7% (16)
Mental Health^c^ (mean (SD))	4.6 (1.0)	4.6 (0.9)	4.7 (0.9)	4.4 (1.1)
Barriers to participation (mean (SD))	1.9 (1.0)	1.8 (1.0)	1.8 (0.9)	2.1 (1.0)

Abbreviations: IMD - Index of Multiple Deprivation,

^a^Participated in one or more NHS-DPP sessions (including initial assessment) or waiting to start

^b^Analysed as a continuous variable

^c^MHI5: a higher score indicates better mental health

### Psychosocial characteristics - results of factor analysis

Confirmatory factor analysis of the 14 psychosocial items generated for the study revealed that the items did not group in line with the hypothesized five factors. Subsequent exploratory factor analysis resulted in four highly reliable factors plus one weakly reliable factor (Additional File 1 Supplement B). These were a reasonable match for key elements of Roger’s Protection Motivation Theory (PMT) [42], which considers factors found to be important in translating information from risk communications into intentions to take action to reduce the threat. We adopted this framework to aid subsequent interpretation and labelled our factors in line with the PMT as: response efficacy (i.e. perceived efficacy of the DPP); vulnerability; response costs; severity, and self-efficacy for reducing T2DM risk. Full descriptions of each of these are given in the Additional File 1 Supplement B.

### Psychosocial characteristics – descriptive statistics

Respondents generally scored high (above 60, out of 100) on the psychosocial factors of generalised self-efficacy, response efficacy, severity and self-efficacy for T2DM risk (Table 2). Vulnerability scores were a little lower (mean of 49.4), and response cost scores lower still (mean of 38.5). Correlations between the psychosocial factors were all below 0.4 (Table B3), with the exception of response efficacy with vulnerability (r = 0.45), implying a reasonable degree of independence.

**Table 2:** Mean scores (SD) on psychosocial subscales by patient subgroup

	All	Participant	Non-participant	No recall of referral
General self-efficacy	64.8 (16.9)	64.6 (18.7)	65.6 (15.9)	63.9 (15.9)
Vulnerability to T2DM	49.4 (15.5)	53.4 (14.8)	46.7 (16.1)	49.5 (15.3)
Severity	76.1 (20.4)	78.8 (21.0)	75.5 (19.50	73.4 (20.5)
Self-efficacy for diabetes risk	76.0 (14.4)	80.4 (14.9)	74.3 (14.2)	72.5 (12.8)
Response efficacy	62.1 (17.5)	69.1 (18.4)	57.1 (15.2)	61.0 (16.2)
Response cost	38.4 (13.3)	34.5 (13.1)	39.9 (13.7)	41.1 (11.6)

Scores are on a scale of 0 to 100, higher scores indicate greater agreement

Abbreviations: T2DM – Type 2 Diabetes Mellitus

### Participation in the NHS-DPP

Of the 325 respondents, 115 (35%) had attended one or more NHS-DPP sessions or were waiting to start, 112 (34%) recalled being invited but did not attend and 98 people (30%) stated that they received no invitation to participate in DPP, despite being coded by their GP as having been invited. Table 1 provides a summary of patient characteristics for each sub-group. The most common reasons for not taking up the offer of a place were (Table A2) feeling no need to attend (33%; n = 37), being undecided (23%; n = 26), too many other demands on time (15%; n = 17), or the place or time being inconvenient (14%; n = 16). Of the 115 who took up the DPP, 13 (11.3%) were waiting to start; 43 (37.4%) were currently attending; 23 (20%) had completed and 36 (31.3%) had left before completion (Table A3). Those who did not recall being invited were similar to the non-participants in terms of age, deprivation, educational level, health literacy (Table 1) and the psychosocial subscales (Table 2), but were more likely to be female, have poor health and not be in employment.

### Relationships between patient characteristics and NHS-DPP uptake

Table 3 summarises the univariable and multivariable analyses comparing NHS-DPP participants and non-participants (excluding those stating they were never invited). Under univariable analysis, uptake was related to older age (OR = 1.03 [1.0 to 1.06], p = 0.050) and not being in work (OR = 2.31 [1.17 to 4.54], p = 0.016), plus the four psychosocial factors of vulnerability (OR = 2.14 [1.26 to 3.5], p = 0.005), self-efficacy for T2DM risk (OR = 1.88 [1.09 to 3.24], p = 0.022), response efficacy (OR = 2.98 [1.82 to 4.90], p < 0.001), and response costs (OR = 0.39 [0.21 to 0.73], p = 0.003) There was no evidence for a relationship to any health-related factor. Backwards stepwise multivariable regression resulted in a model consisting of four factors: age (OR = 1.05 [1.01 to 1.08], p = 0.005) plus the psychosocial factors of vulnerability (OR = 2.53 [1.25 to 5.15], p = 0.010), self-efficacy for T2DM risk (2.38 [1.30 to 4.37], p = 0.005) and response efficacy (OR = 2.23 [1.29 to 3.86], p = 0.004). After accounting for these factors, employment status and severity were no longer significantly associated with uptake.

**Table 3:** Univariate and multivariate analysis of associations with NHS-DPP participation

	Univariable analyses			Multivariable analysis		
	OR	95% CI	p-value	OR	95% CI	p-value
**Demographic factors**						
Gender (P^a^)			0.935			
Male (reference)	-	-				
Female	0.97	(0.52, 1.82)				
Age (P)	1.03	(1.00, 1.06)	0.050*	1.05	(1.01, 1.08)	0.005*
IMD decile (E) (higher is less deprived)	1.08	(0.96, 1.22)	0.208			
Ethnicity (P)			0.363			
White (reference)	-	-				
Non-white	1.49	(0.63, 3.52)				
Living situation (E)			0.525			
Live alone (reference)	-	-				
With one other	0.77	(0.34, 1.74)				
With two or more	0.44	(0.17, 1.13)				
Highest qualification (P)			0.172			
None, GCSE,O,CSE (reference)	-	-				
A level, Degree, other	1.55	(0.83, 2.90)				
Employment (E)			0.016*			
Working (reference)	-	-				
Not working	2.31	(1.17, 4.54)				
**Health factors**						
General Health (N)	1.00	(0.99, 1.01)	0.893			
Mental Health (N)	1.00	(0.98, 1.01)	0.753			
Help on health literacy (P)	1.06	(0.78, 1.43)	0.709			
Known someone with T2DM (P)			0.268			
Yes family (reference)	-	-				
Yes non-family	0.68	(0.34, 1.35)				
No or unsure	0.54	(0.22, 1.35)				
Ever joined another health group (P)			0.603			
Yes (reference)	-	-				
No	0.83	(0.40, 1.69)				
Barriers to participation (E)	1.0	(0.98, 1.02)	0.771			
**Psychosocial factors**						
General self-efficacy (P)	0.99	(0.98, 1.01)	0.592			
Vulnerability (P)	2.14	(1.26, 3.65)	0.005*	2.53	(1.25, 5.15)	0.010*
Severity (P)	1.18	(0.80, 1.74)	0.397			
Self-efficacy for T2DM risk (P)	1.88	(1.09, 3.24)	0.022*	2.38	(1.30, 4.37)	0.005*
Response efficacy (N)	2.98	(1.82, 4.90)	< 0.001*	2.23	(1.29, 3.86)	0.004*
Response cost (P)	0.39	(0.21, 0.73)	0.003*			

Abbreviations: IMD – Index of Multiple Deprivation, T2DM – Type 2 Diabetes Mellitus

^a^P=pre-disposing factor, E = enabling factor, N = need factor. *= p-value < 0.05

Table 4 summarises measures of explanatory power for the regression models using the subsets of demographic, health and psychosocial factors. The psychosocial subset had the highest explanatory power, with an AUC of 0.754 and relatively low AIC of 1.241, Demographic factors were rather weaker (AUC = 0.663; AIC = 1.453), and the subset of health factors were only a little better than chance (AUC = 0.564; AIC = 1.453). A model incorporating all three subsets together performed slightly better than the psychosocial factors alone in terms of AUC (0.802), but the increased AIC (1.323) suggests that this was mainly due to increased complexity. The four-variable backwards stepwise regression model performed very similar to the full model (AUC = 0.778), but the parsimony of this model resulted in a better AIC.

**Table 4:** Summary of measures of explanatory power for logistic regression models (n = 218)

Model	MZ R2	AUC	AIC	Nested model comparison (likelihood-ratio test)	
Demographic factors only	0.102	0.663	1.431	NA	
Health factors only	0.019	0.564	1.453	NA	
Psychosocial factors only	0.252	0.754	1.241	NA	
Demographics + health	0.142	0.696	1.474	Chi2 = 6.56, df = 6 p = 0.363^a^	
Demographics + health + psychosocial	0.389	0.802	1.323	Chi2 = 42.9, df = 1 p < 0.000^a^	
Backwards stepwise regression solution (4 variables)	0.308	0.778	1.193	NA	

Abbreviations: MZ R2 - McKelvey & Zaviana’s pseudo r-squared, AUC - Area under the receiver operating curve, AIC - Akaike Information Criterion (a lower value is preferred)

^a^Compared to demographic factors only model

**Fig. 1:**
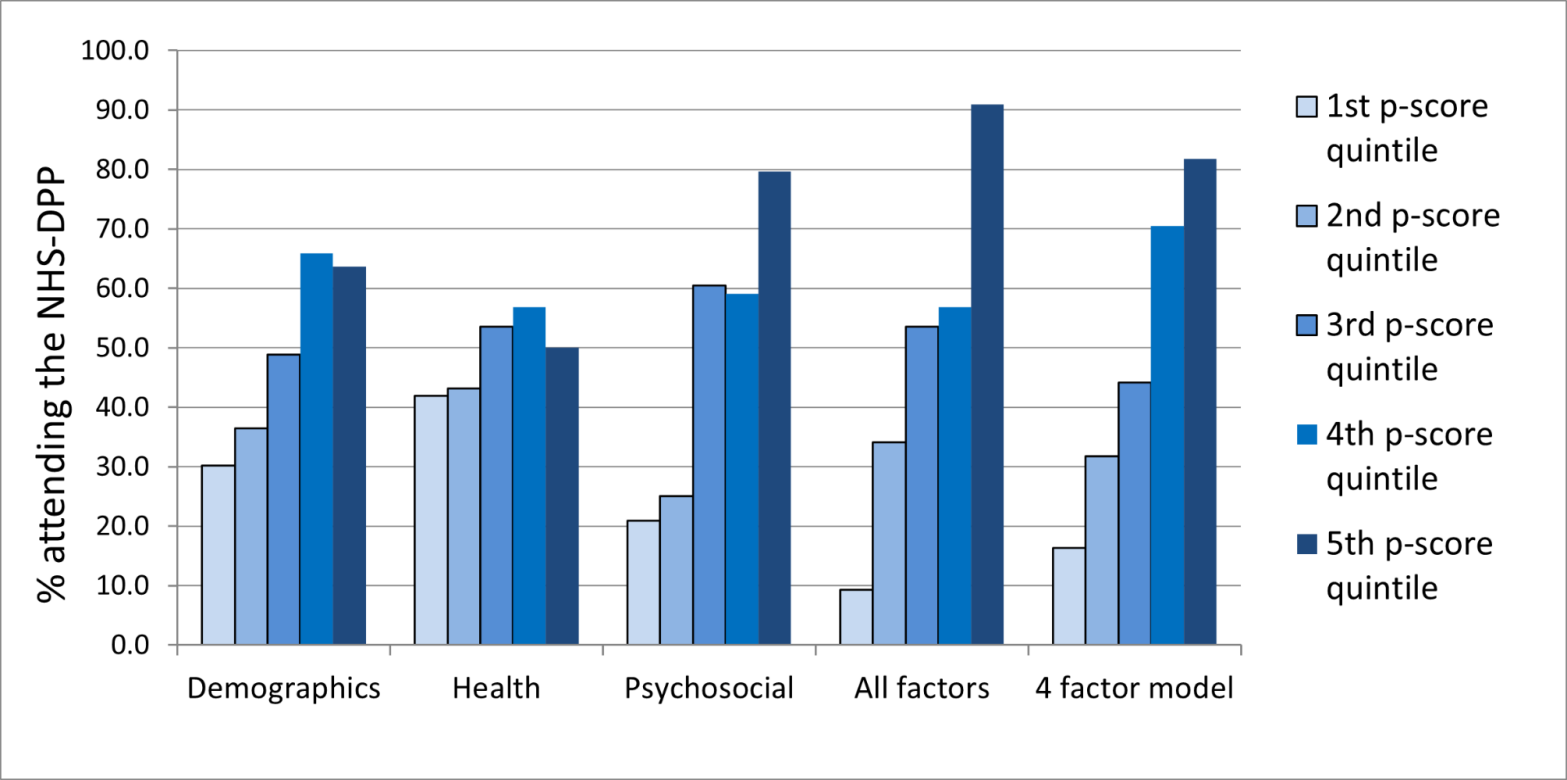
Percentages of patients participating in DPP by propensity score quintile, for different regression models

Table A5 and Fig. 1 present rates of NHS-DPP uptake for individuals in each quintile of propensity scores, for the models described above. For the model consisting of demographic factors alone, the rate of uptake amongst patients in the highest quintile of propensity scores was a little greater than double the rate amongst those in the lowest quintile (64% versus 30%). The 4-factor model consisting of age plus vulnerability, self-efficacy for T2DM risk and response efficacy showed a fivefold increase in uptake (82% versus 16%).

## Discussion

Only a third of patients with a GP record of referral took up the offer of a place on the NHS-DPP. The other two-thirds either declined the offer or could not recall the invitation. Demographic factors on their own showed a modest association with uptake, whilst health factors showed low association. In contrast, psychosocial factors were most strongly associated with uptake. Stepwise regression resulted in a four-variable model with the highest explanatory ability, consisting of age, vulnerability to T2DM, self-efficacy for T2DM risk and response efficacy, all with reasonably distinct relationships to uptake.

The original behavioural framework chosen for the study related to healthcare utilisation, [15, 16] but that model did not fit well to our data. A better fit was with a framework around taking action to reduce risk [42], suggesting that the psychosocial factors at play in deciding about participation in the NHS-DPP were linked to beliefs about risk rather than issues related to healthcare use. This may be related to the fact that the NHS-DPP, although nested within primary care, is a preventative intervention offered by external providers, rather than an NHS delivered healthcare service for a long-standing condition.

The results suggest a dominant role for psychosocial factors with demographic and health factors playing only a minor role. We collected a wide range of demographic and health characteristics and do not believe we overlooked any major factors. The results highlight the limitations of studies that have utilised routinely collected data on demographic variables only, and raise the possibility that psychosocial factors may account for much of the reported differences in uptake between demographic subgroups. However, our sample is too small to draw definite conclusions in this respect. Unlike fixed demographic characteristics, psychosocial perceptions may be amenable to change and could potentially offer a means for increasing uptake.

### Strengths and limitations

The survey response rate was 54%, which although high compared to most surveys in primary care, leaves our results at risk of influence from self-selective response. The survey was restricted to patients invited onto DPP via a letter from their GP. Nationally these accounted for around 40% of patients taking up the DPP over the first 18 months of the programme, with the rest initiated by a face-to-face consultation [12]. The role of psychosocial factors in a face-to-face referral may differ.

Compared to the full cohort of patients with NDH in England in 2019-20 our survey sample included slightly more males (50.5% vs. 48%), were a little older (59% aged 65 or over, vs. 52%), and had higher representation of people from the most and least deprived areas [43]. However, the sample was closely representative of patients with NDH at the surveyed practices (Table A4). Our sample was not large enough to assess whether some patient groups face particular obstacles to uptake, or whether differences exist between areas of England.

The psychosocial items did not group into factors as initially hypothesised. However, an inductively derived solution fitted the data, and aligned with established theory used to understand the impact of risk communications: Protection Motivation Theory. This solution had good reliability coefficients for 4 out of 5 factors. Correlations between the factors were low to moderate, suggesting that they tapped distinct concepts. Two items taken from the Patient Activation Measure [39] loaded with other items to form the self-efficacy for T2DM risk factor. It is important to note that we have not sought to quantify patient activation and the factors we constructed cannot be interpreted in that way.

We were unable to survey patients prior to their decision on NHS-DPP uptake. Non-participants may have rationalised their decision via processes such as cognitive dissonance and self-justification (47); whereas participants may have been influenced in the opposite direction by exposure to the course itself. These processes may have inflated estimated relationships to psychosocial factors. Conversely, due to space limitations other potentially important psychosocial factors may be under-represented, such as family support and social norms around care-seeking. Other factors that were not assessed included BMI, income, attitudes to exercise and diet, and pre-existing knowledge about T2DM. The exclusion of these was principally due to concerns about research burden and avoidance of sensitive topics which might negatively impact on response.

The survey was cross-sectional hence cannot inform on associations with behaviour change or subsequent conversion to T2DM. Nevertheless, wider research evidence has demonstrated that the psychosocial factors we identified play a role in other lifestyle interventions in helping individuals attempt the behavioural changes necessary to reduce their risk [44].

Our outcome was uptake of the initial NHS-DPP assessment. Patients could be at any stage when they completed our questionnaire, hence data on subsequent participation was partial. High attrition during the course results in less than one-fifth of all referrals receiving a required dose (60% or more of the full course) [13]. It seems probable that the psychosocial factors affecting uptake will also influence retention, but in a dynamic fashion related to individual experience of the programme.

### Relationship to existing research

Analysis of the first 300,000 individuals referred to the NHS-DPP nationally found that 47% attended the initial assessment [13]. This rate ignores those who declined referral at consultation or did not respond to a letter (comprising nearly a third of our sample), hence uptake amongst those offered the programme may be considerably lower. Common reactions to a referral letter to the NHS-DPP include shock and confusion at being informed of pre-diabetic status, believing pre-diabetes to be a “non-emergency”, not wanting to be lectured about lifestyle, and viewing other health or social issues as more pressing [18]. Our study also concurs with an analysis of the first 100,000 referrals in finding uptake to increase with age and with area affluence [12]. Low participation rates have been reported of DPPs in other countries. Of more than 2,000 pre-diabetic patients invited by a physician-approved letter into a community-based DPP in Canada just 12% contacted the programme [45]; while within two large health systems in the US only 28% of referrals to a DPP attended one session or more [46]. Similar results have emerged from the US nationally representative National Health Interview Surveys [47].

Large numbers of empirical studies based on a variety of theoretical models have supported the influence of psychosocial factors on a wide range of health behaviours [48–51]. Amongst participants in a Canadian diabetes education programme, the main reasons for withdrawal were practical barriers and psychosocial factors including confidence in knowledge and ability to manage, apathy towards T2DM education, low perceived seriousness and low prioritisation [17]. In meta-analysis, the factors most associated with take-up of a preventative behaviour were perceptions around benefits and barriers, both practical and psychosocial, with weaker effects for outcome severity and personal vulnerability, though effects varied highly across studies [52]. Other research has established that it is possible to change risk perceptions and thereby intentions and behaviour. Randomised trials within a breast cancer screening programme found that women informed they were at increased risk were significantly more likely to join and remain in a lifestyle programme and to lose more weight [53]. More broadly, a large-scale meta-analysis of empirical risk appraisal studies concluded that altered risk perceptions impact on intentions to change behaviour and on changes in behaviour itself, with the largest effect sizes observed when risk appraisals, response efficacy, and self-efficacy were simultaneously heightened [44].

### Implications for practice

Our results suggest that targeting three key areas of patient beliefs could potentially increase NHS DPP uptake from current low levels: first, increasing patient understanding of their vulnerability to T2DM and its potentially severe consequences; second, improving patient self-belief in their ability to carry out and sustain behaviours necessary to reduce this risk; and third, encouraging confidence that the programme can help instil the understanding and skills required. These beliefs could be targeted through messaging from primary care at the point of referral, reinforced by general practice staff knowledgeable in T2DM and the NHS-DPP, and promoted within the content of the programme itself. Mass and social media communication focussed on these three beliefs could also be beneficial [54]. The recently introduced digital version of the NHS DPP could help address the particularly low uptake amongst younger adults [55].

### Implications for research

Means for targeting psychosocial factors and thereby encouraging sustained behaviour change are under-developed [49, 56]. Research is needed on how psychosocial factors might be incorporated into NHS-DPP recruitment processes and promotional materials to increase recruitment. Similarly, how to improve retention by addressing patient perceptions around T2DM risk and self-efficacy within the content of the programme itself. Also needed is a broader re-assessment of content and delivery methods, where research with NHS-DPP participants has linked low satisfaction and attrition to issues such as didactic forms of delivery [57], use of educational content unlikely to change behaviour and under-delivery of more evidence-based content [58], and unclear content [59].

Psychosocial factors may have a different impact at different ages and in different cultures. Research is warranted into the complex relationships between psychosocial factors and sociodemographic characteristics and whether targeting the former can help reduce differential uptake of behavioural change interventions across sociodemographic subgroups.

Our results suggest that a behavioural framework based on taking action to reduce risk is more appropriate for understanding uptake of diabetes prevention programmes - and possibly other preventative health services - than is a framework based on utilisation of healthcare services, for example for long-standing health conditions. Future research could fruitfully explore the relative merits of these two theoretical approaches in relation to differing types of healthcare need.

## Conclusion

Unlike fixed demographic characteristics, psychosocial perceptions may be amenable to change. NHS-DPP uptake rates may be improved by targeting the beliefs of patients about their risk of developing T2DM, their ability to reduce that risk, and the efficacy of the NHS-DPP in helping prevent T2DM, and by improving access to a younger population. Such changes could help facilitate proportional access from across different demographic strata.

## Electronic supplementary material

Below is the link to the electronic supplementary material.

Supplementary Material 1
Additional tables and details of the psychosocial scales. Supplement A: Table A1 Questionnaire items relating to practical barriers to programme participation. Table A2 Breakdown of reasons given for not taking up a place on the NHS DPP. Table A3 Participation status at time of questionnaire completion. Table A4 Comparison of study sample and national diabetes audit data on patients with NDH. Table A5 Rates of NHS-DPP uptake by quintiles of propensity scores. Supplement B: details of the evaluation and modification of the psychosocial scales.

Supplementary Material 2
Patient Survey Questionnaire.

## Data Availability

The dataset generated during the current study is available from the corresponding author on reasonable request.

## List of abbreviations

AIC: Akaike Information Criterion
AUC: Area under the (receiver operating) curve
BMHSU: Behavioral Model of Health Services Utilisation
COPD: Chronic obstructive pulmonary disease
DIPLOMA: DIabetes Prevention – LOng term Multimethod Assessment
GP: General Practice/Practitioner
IMD: Index of Multiple Deprivation
NDH: Non-diabetic hyperglycaemia
NHS-DPP: National Health Service Diabetes Prevention Programme
OR: Odds-ratio
PCN: Primary Care Network
PMT: Protection Motivation Theory
TPB: Theory of Planned Behaviour
T2DM: Type 2 Diabetes Mellitus
