# Supplementary material for: The influence of demographic, health and psychosocial factors on patient uptake of the English NHS diabetes prevention programme
Source: BMC Health Serv Res. 2023 Apr 11;23:352. doi: 10.1186/s12913-023-09195-z (PMC10091609; doi:10.1186/s12913-023-09195-z)
Supplement: Supplementary file 1 — Supplementary Material 1 Additional tables and details of the psychosocial scales. Supplement A: Table A1 Questionnaire items relating to practical barriers to programme participation. Table A2 Breakdown of reasons given for not taking up a place on the NHS DPP. Table A3 Participation status at time of questionnaire completion. Table A4 Comparison of study sample and national diabetes audit data on patients with NDH. Table A5 Rates of NHS-DPP uptake by quintiles of propensity scores. Supplement B: details of the evaluation and modification of the psychosocial scales. [file 12913_2023_9195_MOESM1_ESM.docx]

**Additional file 1**

**Supplement A: additional tables**

| **Table A1 Questionnaire items relating to practical barriers to programme participation** |
| --- |
| I have difficulties using health services due to language and culture |
| My other health problems or disability are more of a priority than diabetes prevention |
| I have enough time to take care of my own health |
| I am satisfied with my ability to perform my daily living activities* |
| I have enough money to meet my needs* |
| I am able to get around well* |
| *Item from the WHO-QOL |

| **Table A2 Breakdown of reasons given for not taking up a place on the NHS DPP** | |
| --- | --- |
| Too many other demands on time | 15.2% (17) |
| Place or time of course inconvenient | 14.3% (16) |
| Was undecided and didn’t enquire further | 23.2% (26) |
| Felt no need to attend | 33.0% (37) |
| Felt program not suitable for me | 8.0% (9) |
| Other (open-ended item):  Admin error  Well or blood sample “OK”  Too ill to attend | 4.5% (5)  8.9% (10)  3.6% (4) |

| **Table A3 Participation status at time of questionnaire completion*** | |
| --- | --- |
| Waiting to start the NHS-DPP | 11.3% (13) |
| Currently attending | 37.4% (43) |
| Completed | 20.0% (23) |
| Left the course before completion | 31.3% (36) |
| Reasons given for leaving**:  Other things got in the way  Not learning anything new  Decided not for me  Other^:  Admin error, trainer issue or content  Well or blood sample “OK”  Too ill to continue  Transport problem | 16.5% (19)  7.8% (9)  3.5% (4)  6.1% (7)  2.6% (3)  1.7% (2)  1.7% (2) |
| *Based on participants only  **More than one reason could be given  ^Based on responses to a free-text item | |

| **Table A4 Comparison of study sample and national NDA data on patients with NDH** | | | |
| --- | --- | --- | --- |
|  | This study | NDA – patients with NDH, practices in our sample* | NDA – patients with NDH, all England* |
| Gender | | | |
| Male | 50.5% (162) | 49.3% (4,120) | 48.0% (1,027,645) |
| Female | 49.5% (159) | 50.7% (4,240) | 52.0% (1,112,435) |
| Mean age (SD) | 64.8% (14.3) | - | - |
| % 65 or over | 58.2% (188) | 53.3% (4,455) | 51.7% (1,106,395) |
| IMD | | | |
| 1^st^ quintile | 25.5% (83) | 24.1% (2.010) | 21.2% (454,520) |
| 2^nd^ | 20.9% (68) | 18.6% (1,555) | 20.7% (443,170) |
| 3^rd^ | 11.1% (36) | 13.9% (1,160) | 20.3% (434,140) |
| 4^th^ | 19.4% (63) | 20.2% (1,685) | 19.3% (413,185) |
| 5^th^ | 23.1% (75) | 23.6% (1,970) | 18.4% (393,840) |
| Ethnicity | | | |
| White | 80.9% (262) | 64.6% (5,395) | 67.1% (1,435,055) |
| Other | 19.1% (62) | 13.6% (1,840) | 17.6% (376,355) |
| Unknown | 0.0% (1) | 22.0% (1,840) | 15.4% (328,675) |
| *Source: National Diabetes Audit, Non-Diabetic Hyperglycaemia, 2019- 2020, Diabetes Prevention Programme, Data Release. <https://digital.nhs.uk/data-and-information/publications/statistical/national-diabetes-audit/hyperglycaemia-2019--2020-diabetes-prevention-programme-data-release> | | | |

| **Table A5 Rates of NHS-DPP uptake by quintiles of propensity scores** | | | | | |
| --- | --- | --- | --- | --- | --- |
| Factor subset | 1^st^ quintile | 2^nd^ | 3^rd^ | 4th | 5th |
| Demographic factors | 30.2% | 36.4% | 48.8% | 65.9% | 63.6% |
| Health factors | 41.9% | 43.2% | 53.5% | 56.8 | 50.0 |
| Psychosocial factors | 20.9 | 25.0 | 60.5 | 59.1 | 79.6 |
| Demographics + health | 23.3 | 40.9 | 51.1 | 63.6 | 65.9 |
| Demographics + health + psychosocial | 9.3% | 34.1% | 53.5% | 56.8% | 90.9% |
| Regression solution* | 16.3% | 31.8% | 44.2% | 70.5% | 81.8% |
| * Consisting of age, vulnerability to diabetes, self-efficacy for diabetes risk, and response efficacy | | | | | |

**Supplement B:** **Evaluation and modification of the psychosocial scales**

**Statistical methods**

Generalised self-efficacy was measured using a widely-used validated 4-item scale using the standard scoring method.(1) To evaluate the hypothesised 5-factor latent structure underlying the 14 psychosocial items generated for the study, we applied confirmatory factor analysis (CFA) within a Structural Equation Modelling (SEM) framework using AMOS v23. Then to evaluate alternative structures we first ran exploratory factor analysis (EFA) in SPSS v23 using principal axis factoring with oblimin rotation to allow oblique factors, followed by SEM in AMOS v23 to obtain goodness of fit indices and for modification analysis. In all cases the criteria for a good-fitting model was the combinational rule recommended by Hu and Bentler of a Comparative Fit Index (CFI) approaching 0.95 or above, and Standardized Root Mean Square Residual (SRMR) <=0.09.(2) The question items demonstrated some skew, therefore Omega coefficients were used to assess reliability: omega makes fewer assumptions and is less biased than Cronbach’s Alpha in this situation.(3) An Omega value of 0.7 or higher was taken to indicate good reliability.

Twenty-eight individuals (8.6%) had missing data on one or more of the 14 items making up the 5 novel psychosocial factors. To maximise the sample available for investigating the factor structure we imputed missing item scores for all individuals missing on no more than three items. We used regression imputation to produce single imputed values, based on the scores for all answered items, referral group, general health, gender, age, IMD decile and ethnicity (white versus other). This reduced the number of respondents excluded from the factor analysis to just eight (2.5%). AMOS has the ability to perform regression imputation from variables within the model, but we preferred to do this externally to include these additional factors.

**Results**

Table B1 presents summary results for the 14 psychosocial items and initial hypothesised subscale structure. Omega reliability coefficients were below 0.7 for all subscales with the exception of the Importance of the Programme subscale. In addition, CFA of the 14 items demonstrated a low CFI (0.676) and high RMSEA (0.123).

| **Table B1: Original hypothesised psychosocial factors** | |
| --- | --- |
| Subscale | Omega coefficient |
| 1. Perceptions about the NHS-DPP | |
| The programme can help me reduce my risk of diabetes | 0.72 |
| The diabetes prevention programme couldn't tell me anything new |  |
| I can look after my risk of diabetes without the help of a programme |  |
| 2. Risk associated with T2DM | |
| My risk of developing diabetes is too low to worry about | 0.50 |
| If I carry on as normal, there is a good chance that I will develop diabetes |  |
| Diabetes is not a very serious illness |  |
| 3. Ability to reduce risk | |
| It is too difficult for me to change my lifestyle to reduce my diabetes risk | 0.56 |
| Nothing I do can reduce my risk of getting diabetes |  |
| I can do whatever is needed to reduce my risk of getting diabetes |  |
| 4. Motivation to reduce risk | |
| I am happy with my lifestyle as it is. | 0.2 |
| It is important that I manage my risk of getting diabetes |  |
| Going to this programme requires a lot of effort |  |
| 5. Active role in own health | |
| I am the person responsible for managing my health conditions* | 0.64 |
| Taking an active role in my own healthcare is the most important factor in determining my health and ability to function* |  |
| * From the Active Roles subscale of the Patient Activation Measure | |

We applied limited modifications to the model, based on the AMOS modification indices, but these failed to make an appreciable improvement. We therefore applied exploratory factor analysis (EFA) to examine whether a viable alternative grouping of the items existed with an acceptably coherent factor structure.

Four factors with eigenvalues>1 were identified, we therefore initially examined 3, 4, and 5-factor solutions, from which we retained the 5-factor solution as the most internally consistent, and then used AMOS to examine goodness of fit and refine the model.

In its unmodified form this model had CFI=0.803 and SRMR=0.0976. On the basis of the AMOS modification indices and maintaining logical consistency with the existing items on each factor, four cross-loading paths were added from the latent factors to other items in a stepwise manner, resulting in a final model with CFI=0.95 and SRMR=0.052, meeting our criteria for fit. This model is summarised in table B2 and illustrated in figure B1.

Subsequent to the analysis, it was observed that the resulting factors demonstrated a reasonable match to factors in Rogers’ Protection Motivation Theory (PMT) model.(4) To aid interpretation, the factors have been labelled according to the PMT factor they best correspond to:

Factor 1: Response (programme) efficacy (omega=0.78). Perceived effectiveness of the response (participation in the DPP) in removing or reducing potential harm. Includes a minor contribution from the item “It is important that I manage my risk of getting diabetes”, which although not programme-specific indicates a recognition of need.

Factor 2: Vulnerability (omega=0.72). Perceived likelihood of experiencing harm. Two items - “I am happy with my lifestyle as it is” and “I can do whatever is needed to reduce my risk of getting diabetes”, both negatively loading – implicitly rather than explicitly imply a low expectation of harm.

Factor 3: Response costs (omega=0.76). Costs associated with attending the DPP. Items relate to difficulty in doing what is needed to reduce risk (e.g. changing lifestyle, attending the programme), together with an attitude of low risk.

Factor 4: Severity (omega=0.64). Perceived degree of harm from diabetes. The item “Nothing I do can reduce my risk of getting diabetes” tended to be agreed with by those who regarded diabetes as not serious.

Factor 5: Self-efficacy for reducing risk (omega=0.74). Combines the two PAM active role items with items relating to personal ability to reduce risk.

All factors except Severity (Factor 4) had an omega coefficient of >0.7. However, the Severity factor included only two items and reliability is partly a function of the number of contributing items. Although omega is less biased than alpha when data is skewed, some bias is likely. However, this is typically negative, suggesting that reliability will be under-estimated.(3)

| **Table B2: Final modified factor model, factor loadings and omega values** | | | | |
| --- | --- | --- | --- | --- |
|  | | Original subscale | Factor loading | Omega |
| **Factor 1: Response (programme) efficacy** | | | | |
| 9 | The programme can help me reduce my risk of diabetes | 1 | 0.62 | 0.78 |
| 22 | The diabetes prevention programme couldn't tell me anything new | 1 | -0.72 |  |
| 21 | I can look after my risk of diabetes without the help of a programme | 1 | -0.85 |  |
| *19** | *It is important that I manage my risk of getting diabetes** | 4 | 0.31 |  |
| **Factor 2: Vulnerability** | | | | |
| 10 | I am happy with my lifestyle as it is | 4 | -0.58 | 0.72 |
| 27 | My risk of developing diabetes is too low to worry about | 2 | -0.66 |  |
| 23 | If I carry on as normal, there is a good chance that I will develop diabetes | 2 | 0.64 |  |
| *25** | *I can do whatever is needed to reduce my risk of getting diabetes* | 3 | -0.35 |  |
| **Factor 3: Response costs** | | | | |
| 30 | It is too difficult for me to change my lifestyle to reduce my diabetes risk | 3 | 0.78 | 0.76 |
| 31 | Going to this programme requires a lot of effort | 4 | 0.45 |  |
| *27** | *My risk of developing diabetes is too low to worry about* | 2 | 0.54 |  |
| *21** | *I can look after my risk of diabetes without the help of a programme* | 1 | -0.36 |  |
| **Factor 4: Severity** | | | | |
| 14 | Nothing I do can reduce my risk of getting diabetes | 3 | -0.78 | 0.64 |
| 16 | Diabetes is not a very serious illness | 2 | -0.59 |  |
| **Factor 5: Self-efficacy for diabetes risk** | | | | |
| 19 | It is important that I manage my risk of getting diabetes | 4 | 0.56 | 0.74 |
| 25 | I can do whatever is needed to reduce my risk of getting diabetes | 3 | 0.49 |  |
| 26 | I am the person responsible for managing my health conditions | 5 | 0.70 |  |
| 28 | Taking an active role in my own healthcare is the most important factor in determining my health and ability to function | 5 | 0.67 |  |
| *Italics indicate items cross-loading from other factors | | | | |

**Figure B1: Final modified 5-factor structural equation model**


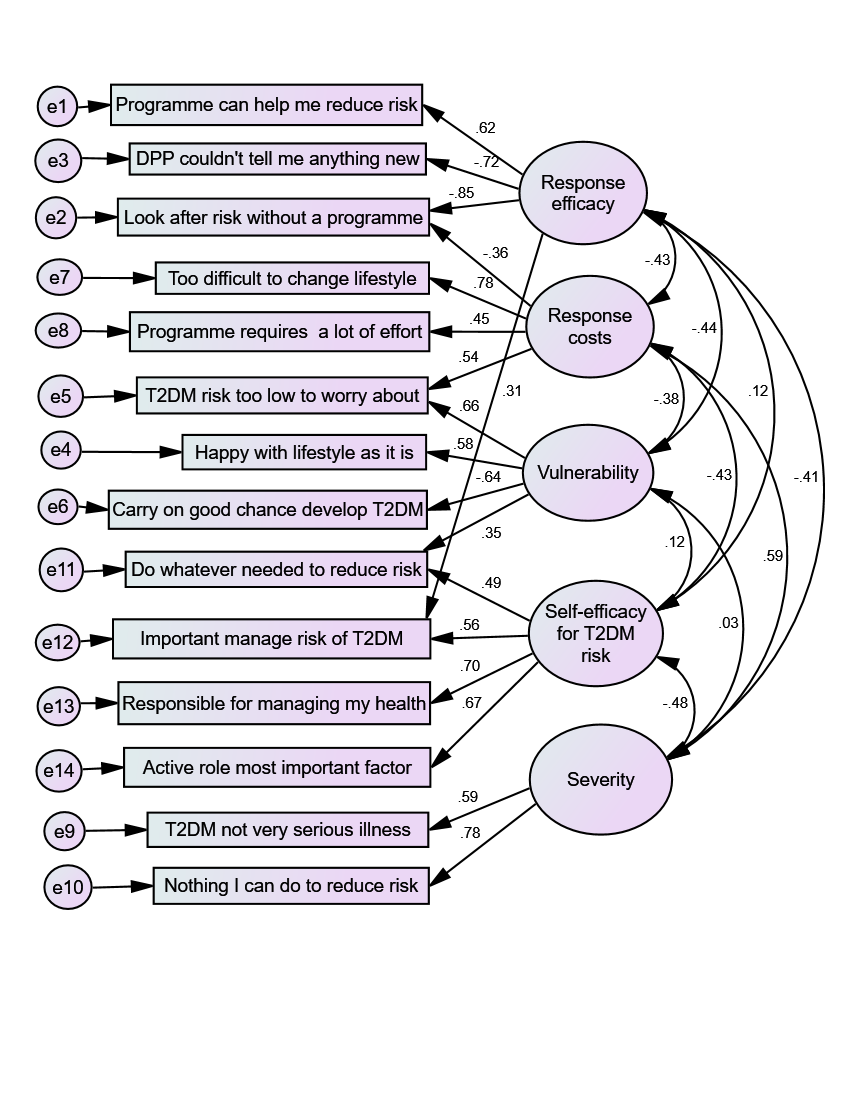


**Factor scores**

To derive scores on each resulting factor for individual patients, we computed a mean score across the items on the factor weighted by the item factor loading. This method was preferred over the factor scores computed by AMOS as it preserves the original item scaling resulting in more directly interpretable scores,(5) whereas factor scores have a standardised mean of zero and standard deviation of 1. For analysis, we then expressed these scores as a percentage, from 0 to 100, of the maximum possible score.

Correlations between the factor scores were mostly low and exceeded 0.4 for only one pair of factors, response efficacy and vulnerability (r=0.45; Table B3).

**Table B3: correlations between the psychosocial factor scores**

|  | Response efficacy | Vulnerability | Response costs | Severity | Self-efficacy for diabetes |
| --- | --- | --- | --- | --- | --- |
| Response efficacy | 1.0 |  |  |  |  |
| Vulnerability | 0.45 | 1.0 |  |  |  |
| Response costs | -0.10 | -0.11 | 1.0 |  |  |
| Severity | 0.24 | 0.14 | -0.39 | 1.0 |  |
| Self-efficacy for diabetes risk | 0.17 | -0.17 | -0.34 | 0.32 | 1.0 |

**References**

1. Tambs K, Røysamb E. Selection of questions to short-form versions of original psychometric instruments in MoBa. Norsk Epidemiologi. 2014;24(1-2).

2. Hu Lt, Bentler PM. Cutoff criteria for fit indexes in covariance structure analysis: Conventional criteria versus new alternatives. Structural Equation Modeling: A Multidisciplinary Journal. 1999;6(1):1-55.

3. Trizano-Hermosilla I, Alvarado JM. Best Alternatives to Cronbach's Alpha Reliability in Realistic Conditions: Congeneric and Asymmetrical Measurements. Frontiers in Psychology. 2016;7.

4. Rodgers WM, Conner M, Murray TC. Distinguishing among perceived control, perceived difficulty, and self-efficacy as determinants of intentions and behaviours. British Journal of Social Psychology. 2008;47(4):607-30.

5. DiStefano C, Zhu M, Mîndrilã D. Understanding and Using Factor Scores: Considerations for the Applied Researcher. Practical Assessment, Research, and Evaluation. 2009;14(1).
