# Supplementary material for: The influence of demographic, health and psychosocial factors on patient uptake of the English NHS diabetes prevention programme
Source: BMC Health Serv Res. 2023 Apr 11;23:352. doi: 10.1186/s12913-023-09195-z (PMC10091609; doi:10.1186/s12913-023-09195-z)
Supplement: Supplementary file 2 — Supplementary Material 2 Patient Survey Questionnaire. [file 12913_2023_9195_MOESM2_ESM.pdf]

MANCHESTER 1824
The University of Manchester

A study about preventing diabetes:
the “Healthier You” evaluation questionnaire

Understanding why people do or do not participate in the NHS **“Healthier You”** diabetes prevention programme is important for improving the service for others. Whether or not you joined the programme your views are important.

In recognition of completing the questionnaire, you can claim a shopping voucher worth £25. Please see the next page for further details.

The questionnaire should take 10 to 15 minutes to complete.

For privacy, your completed questionnaire will be anonymised when we receive it and kept separate from your contact details, both in secure locations.

If you would prefer to complete the questionnaire by phone, please leave a message for the Researcher, Adrine Woodham on **0161-275-7788** or email her on **adrine.woodham@manchester.ac.uk** to arrange a convenient time.

Returning the Questionnaire

Please post your completed questionnaire to The University of Manchester using the FREEPOST envelope provided.

If you DO NOT want to participate, please post the blank questionnaire back to us in the FREEPOST envelope, then we will not send any reminders.

When the research has been completed, a summary will be available on the study website [https://www.clahrc-gm.nihr.ac.uk/projects/diploma-evaluation-national-nhs-diabetes-prevention-programme].

Thank you in anticipation for your help in this research, which will benefit peoples' future health and wellbeing

FOR OFFICE USE: ID:XXX/YYYYYYYY

Your contact details

This questionnaire has been sent to you by your GP. The research team at the University of Manchester does NOT have your contact details. However, for two reasons we ask for your contact details: (1) to send your shopping vouchers; and (2) to invite you to participate in a follow-up questionnaire survey at a future date.

Please circle either **YES** or **NO** to each question below.

1	May we send you shopping vouchers (Love2shop) worth £25, in recognition of your contribution to the research?	**YES / NO**
2	May we send you an invitation at a future date to participate in a short follow-up survey?	**YES / NO**

If you circled **YES** to either of the above, please give your contact details below:

Name: ____

Address: ____

____

____

Phone: ____

EMAIL: ____

If you only ticked YES to the shopping vouchers we will delete your contact details after we have posted the vouchers out to you, otherwise we will keep them for the full duration of the study.

Love2shop vouchers are valid at a wide selection of stores including Boots, TKMaxx, Wilko and Go Outdoors. For full details see https://www.highstreetvouchers.com/gift/where-to-spend-love2shop-vouchers

HEALTHIER YOU survey questionnaire

Please circle one answer for each question. There are no right or wrong answers. If unsure how to answer a question, please give your best guess.

Please circle one answer for each question

1	Have you ever received an invitation to join the Healthier You Diabetes Prevention Programme?	**YES / NO**<br>*If NO, please go to question 6*
2	Was the invitation clear to you?	**YES / PARTIALLY / NO**
3	Did you telephone the number in the letter to book a place or find more information?	**YES / NO / CAN'T REMEMBER**
4	How many Healthier You programme sessions have you been to?	**NONE / 1 / 2 to 5 / 6 or more**
5a	**If you haven't attended any sessions, please circle one option, then go to Question 6. Otherwise, go to 5b below**<br>a. I am waiting to start the programme<br>b. I wanted to join but could not fit the programme around the other demands on my time<br>c. I wanted to join but the meeting place and/or time was not convenient<br>d. I was undecided about joining and never enquired any further<br>e. I did not feel that I needed to attend the programme<br>f. I did not feel that the programme would be suitable for someone like me<br>*Please give details or other reasons if you wish:-*<br>……..	
5b	**If you have attended one or more sessions, please circle one option**<br>a. I am currently attending the Healthier You programme<br>b. I have completed the Healthier You programme<br>c. I started the programme and wanted to complete it but other things got in the way<br>d. I stopped attending the programme because I felt it was not for me<br>e. I stopped attending the programme because I didn't feel I was learning anything new<br>*Please give details or other reasons if you wish:-*<br>……..	
6	Have you ever joined another programme or group for improving health?(eg Weightwatchers, Health Walks group)	**YES / NO**
	If yes, please give details:-	
7	Have you known anyone with diabetes?	**YES / NO / UNSURE**
8	Have any of your parents, brothers or sisters had diabetes?	**YES / NO / UNSURE**

For each statement below, please circle ONE number only, to show how much you agree or disagree. *Some questions may seem similar, but please answer them all*		*Circle the appropriate number* Agree strongly	Agree	Disagree	Disagree strongly
9	The programme can help me reduce my risk of diabetes	1	2	3	4
10	I am happy with my lifestyle as it is	1	2	3	4
11	I am satisfied with my ability to perform my daily living activities	1	2	3	4
12	I have enough money to meet my needs	1	2	3	4
13	My other health problems or disability are more of a priority than diabetes prevention	1	2	3	4
14	Nothing I do can reduce my risk of getting diabetes	1	2	3	4
15	When all said and done I can always solve difficult problems if I try hard enough	1	2	3	4
16	Diabetes is not a very serious illness	1	2	3	4
17	I have difficulties using health services due to language and culture	1	2	3	4
18	I am able to get around well	1	2	3	4
19	It is important that I manage my risk of getting diabetes	1	2	3	4
20	I have enough time to take care of my own health	1	2	3	4
21	I can look after my risk of diabetes without the help of a programme	1	2	3	4
22	The diabetes prevention programme couldn't tell me anything new	1	2	3	4
23	If I carry on as normal, there is a good chance that I will develop diabetes	1	2	3	4

		Circle the appropriate number			
		Agree strongly	Agree	Disagree	Disagree strongly
24	I can remain calm when facing difficulties because I can rely on my coping abilities	1	2	3	4
25	I can do whatever is needed to reduce my risk of getting diabetes	1	2	3	4
26	I am the person responsible for managing my health conditions	1	2	3	4
27	My risk of developing diabetes is too low to worry about	1	2	3	4
28	Taking an active role in my own healthcare is the most important factor in determining my health and ability to function	1	2	3	4
29	I am confident that I can deal efficiently with unexpected events	1	2	3	4
30	It is too difficult for me to change my lifestyle to reduce my diabetes risk	1	2	3	4
31	Going to this programme requires a lot of effort	1	2	3	4
32	If I am in trouble, I can usually think of a solution	1	2	3	4

During the past month….

		Circle the appropriate answer					
		All of the time	Most of the time	A good bit of the time	Some of the time	A little of the time	None of the time
33	Have you been a very nervous person?	1	2	3	4	5	6
34	Have you felt calm and peaceful?	1	2	3	4	5	6
35	Have you felt downhearted and blue?	1	2	3	4	5	6
36	Have you been a happy person?	1	2	3	4	5	6
37	Have you felt so down in the dumps that nothing could cheer you up?	1	2	3	4	5	6

38	In general, would you say your health is: *(please circle one answer)*<br>**Excellent / Very good / Good / Fair / Poor**
39	How often do you need help to understand written material (such as instructions and leaflets from your doctor or pharmacy? *(please circle one)*<br>**Never / Rarely / Sometimes / Often / Always**
40	Which of these best describes your current occupation? *(please circle one)*<br>**a. In paid work (full/part time or self-employed)**<br>**b. Unemployed**<br>**c. Looking after family / home**<br>**d. Voluntary work**<br>**e. Not working due to disability or ill-health**<br>**f. Retired**<br>**g. In education or training**<br>**h. Other, please give details……………………………………………………….**
41	What is your ethnic group? *(please circle one)*<br>**a. White**<br>**b. Mixed or multiple ethnic groups**<br>**c. Black/African/Caribbean/Black British**<br>**d. Asian/Asian British**<br>**e. Other ethnicity**:- *please give details below*<br>…………………………………………………………………………………………………
42	What is your highest qualification? *(please circle one)*<br>**None / GCSE, O level, CSE / A level / Degree**<br>Other qualifications…………………………………………………………………………..
43	What is your gender? *(Please write below)*<br>…………………………………………………………………………………………
44	How old are you?..............................years
45	How many adults do you live with? …………………………………………<br>How many children do you live with?...

If you have any have queries or problems, please leave a message for the Researcher at University of Manchester, Adrine Woodham on Tel: 0161-275-7788 or email adrine.woodham@manchester.ac.uk

Please post this questionnaire back to us in the prepaid envelope.

Thank you.
